# Pharmacological Modulation of the Wnt/β-Catenin Pathway Inhibits Proliferation and Promotes Differentiation of Long-Lived Memory CD4^+^ T Cells in Antiretroviral Therapy-Suppressed Simian Immunodeficiency Virus-Infected Macaques

**DOI:** 10.1128/JVI.01094-19

**Published:** 2019-12-12

**Authors:** M. Mavigner, M. Zanoni, G. K. Tharp, J. Habib, C. R. Mattingly, M. Lichterfeld, M. T. Nega, T. H. Vanderford, S. E. Bosinger, A. Chahroudi

**Affiliations:** a Department of Pediatrics, Emory University School of Medicine, Atlanta, Georgia, USA; b Emory Vaccine Center, Emory University, Atlanta, Georgia, USA; c Yerkes National Primate Research Center, Emory University, Atlanta, Georgia, USA; d Ragon Institute of MGH, MIT and Harvard, Cambridge, Massachusetts, USA; e Harvard Medical School, Boston, Massachusetts, USA; f Emory + Children’s Center for Childhood Infections and Vaccines, Children’s Healthcare of Atlanta, Atlanta, Georgia, USA; Icahn School of Medicine at Mount Sinai

**Keywords:** PRI-724, SIV, Wnt/β-catenin pathway, memory CD4^+^ T cells, proliferation, stemness

## Abstract

The major obstacle to human immunodeficiency type 1 virus (HIV-1) eradication is a reservoir of latently infected cells that persists despite long-term antiretroviral therapy (ART) and is maintained through cellular proliferation. Long-lived memory CD4^+^ T cells with high self-renewal capacity, such as central memory (CM) T cells and stem cell memory (SCM) T cells, are major contributors to the viral reservoir in HIV-infected individuals on ART. The Wnt/β-catenin signaling pathway regulates the balance between self-renewal and differentiation of SCM and CM T cells, and pharmacological manipulation of this pathway offers an opportunity to interfere with the proliferation of latently infected cells. Here, we evaluated *in vivo* a novel approach to inhibit self-renewal of SCM and CM CD4^+^ T cells in the rhesus macaque (RM) model of simian immunodeficiency (SIV) infection. We used an inhibitor of the Wnt/β-catenin pathway, PRI-724, that blocks the interaction between the coactivator CREB-binding protein (CBP) and β-catenin, resulting in the cell fate decision to differentiate rather than proliferate. Our study shows that PRI-724 treatment of ART-suppressed SIV_mac251_-infected RMs resulted in decreased proliferation of SCM and CM T cells and modified the SCM and CM CD4^+^ T cell transcriptome toward a profile of more differentiated memory T cells. However, short-term treatment with PRI-724 alone did not significantly reduce the size of the viral reservoir. This work demonstrates for the first time that stemness pathways of long-lived memory CD4^+^ T cells can be pharmacologically modulated *in vivo*, thus establishing a novel strategy to target HIV persistence.

**IMPORTANCE** Long-lasting CD4^+^ T cell subsets, such as central memory and stem cell memory CD4^+^ T cells, represent critical reservoirs for human immunodeficiency virus (HIV) persistence despite suppressive antiretroviral therapy. These cells possess stem cell-like properties of enhanced self-renewal/proliferation, and proliferation of latently infected memory CD4^+^ T cells plays a key role in maintaining the reservoir over time. Here, we evaluated an innovative strategy targeting the proliferation of long-lived memory CD4^+^ T cells to reduce viral reservoir stability. Using the rhesus macaque model, we tested a pharmacological inhibitor of the Wnt/β-catenin signaling pathway that regulates T cell proliferation. Our study shows that administration of the inhibitor PRI-724 decreased the proliferation of SCM and CM CD4^+^ T cells and promoted a transcriptome enriched in differentiation genes. Although the viral reservoir size was not significantly reduced by PRI-724 treatment alone, we demonstrate the potential to pharmacologically modulate the proliferation of memory CD4^+^ T cells as a strategy to limit HIV persistence.

## Abstract (precis)

Long-lasting CD4^+^ T cell subsets, such as central memory and stem cell memory CD4^+^ T cells, represent critical reservoirs for human immunodeficiency virus (HIV) persistence despite suppressive antiretroviral therapy. These cells possess stem cell-like properties of enhanced self-renewal/proliferation, and proliferation of latently infected memory CD4^+^ T cells plays a key role in maintaining the reservoir over time. Here, we evaluated an innovative strategy targeting the proliferation of long-lived memory CD4^+^ T cells to reduce viral reservoir stability. Using the rhesus macaque model, we tested a pharmacological inhibitor of the Wnt/β-catenin signaling pathway that regulates T cell proliferation. Our study shows that administration of the inhibitor PRI-724 decreased the proliferation of SCM and CM CD4^+^ T cells and promoted a transcriptome enriched in differentiation genes. Although the viral reservoir size was not significantly reduced by PRI-724 treatment alone, we demonstrate the potential to pharmacologically modulate the proliferation of memory CD4^+^ T cells as a strategy to limit HIV persistence.

## INTRODUCTION

The key obstacle to cure human immunodeficiency virus (HIV) infection is a reservoir of latently infected memory CD4^+^ T cells that persists despite long-term antiretroviral therapy (ART) and causes a rebound of viremia if therapy is interrupted. The reservoir of latently infected memory CD4^+^ T cells that persists on long-term ART has been shown to include mostly central memory (CM), transitional memory (TM), and the more recently identified stem cell memory (SCM) CD4^+^ T cells (1, 2). Interestingly, SCM CD4^+^ T cells have been shown to disproportionally contribute to the total HIV reservoir in patients on long-term ART despite their small contribution to the overall CD4^+^ T cell pool (1, 3). Furthermore, a lower frequency of latently infected SCM and/or CM CD4^+^ T cells has been found in adult (4, 5) and pediatric nonprogressors (6), as well as in a cohort of patients treated soon after infection who exhibited controlled viremia after ART discontinuation (termed “post‐treatment HIV controllers” [7, 8]). Due to their stem cell-like properties of enhanced self-renewal/proliferation, SCM and CM CD4^+^ T cells may provide a more stable reservoir for HIV than effector memory (EM) T cells, which are more susceptible to programmed cell death (2, 9).

Accumulating reports suggest that proliferation of infected cells plays a critical role in maintaining the viral reservoir (10–13). Clonal expansion of infected cells was first suggested by the repeated detection of identical sequences among residual plasma viruses in individuals on ART (14, 15). More recently, studies using integration site analysis and/or near full-length single-genome proviral sequencing demonstrated that HIV-infected cells can proliferate in individuals on ART by identifying expanded cellular clones carrying provirus integrated into a unique site in the human genome (16–21). While the mechanisms driving clonal expansion of infected cells are currently unknown, these studies suggest that homeostatic proliferation of long-lived memory CD4^+^ T cells could be key to this process (22–24). According to the linear developmental model, memory CD4^+^ T cells display a cellular hierarchy, with SCM CD4^+^ T cells giving rise to successively more differentiated T cell lineages that include CM, TM, and EM CD4^+^ T cells (25–27). The long-lived SCM T cells and, to a lesser extent, CM T cells display stem cell-like characteristics, continually maintaining their own pool size through homeostatic proliferation (1, 28–31). Control of the two distinct fates of SCM and CM T cells, i.e., self-renewal versus differentiation, is regulated by molecular stem cell-like pathways, which include the Wnt/β-catenin signaling pathway (29, 30, 32, 33).

The Wnt/β-catenin pathway has been identified as a key driver for the homeostasis of stem cells and has also been shown to control the generation and self-renewal of memory T cells, specifically of SCM T cells, in mice and humans (29, 30, 32, 34). The Wnt/β-catenin pathway involves a complex cascade of multiple proteins that interact in the cytoplasm and nucleus to result in transcription of specific genes involved in stem cell biology (35). In the final stages of this pathway, β-catenin enters the nucleus and forms a complex with members of the T cell factor (TCF) family of transcription factors that become active by the interaction with a transcriptional coactivator. The transcriptional coactivator p300, which promotes cell differentiation, and its homologue CREB-binding protein (CBP), which promotes self-renewal, are bimodal regulators of TCF/β-catenin-mediated transcription (36). The critical decision by β-catenin to utilize either CBP or p300 thus guides the cell to either proliferate/maintain potency or to initiate a differentiation transcriptional program, respectively. Pharmacological manipulation of this pathway has been the subject of extensive research in oncology, with the primary goal of inhibiting the proliferation of cancer stem cells, a small group of cancer cells that persist despite classical antineoplastic therapy. Among the antagonists of Wnt signaling emerging from this work, the small molecule PRI-724 has been demonstrated to specifically inhibit the CBP/β-catenin interaction, thereby selectively blocking the major pathway involved in cell self-renewal while conversely promoting cell differentiation (37–40).

Here, we assessed the impact of Wnt pathway modulation on the proliferation of the long-lived SCM and CD4^+^ T cells using the well-established model of simian immunodeficiency virus (SIV) infection of rhesus macaques (RMs). This *in vivo* study included 12 SIV-infected RMs in which virus replication was effectively suppressed with a potent, three-drug ART regimen to investigate the effect of PRI-724 administration for a 12-week period. In this preclinical experimental setting, we found that PRI-724 was safe, reduced SCM and CM CD4^+^ T cell proliferation, and induced changes in the transcriptomic profile of the SCM and CM CD4^+^ T cells that were indicative of cell differentiation but did not alter the viral reservoir of latently infected CD4^+^ T cells. This study suggests that targeting the Wnt/β-catenin pathway is a novel approach to limit proliferation of memory CD4^+^ T cells that may be complementary to strategies to reduce HIV/SIV persistence in long-lived reservoirs.

## RESULTS

### Experimental design.

**FIG 1:**
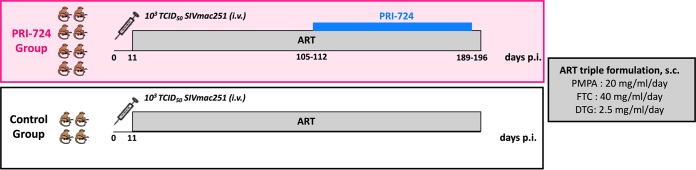
Experimental study design. Twelve rhesus macaques (RMs) were infected i.v. with 1,000 50% tissue culture infective dose (TCID_50_) of SIV_mac251_. Starting day 11 postinfection (p.i.), RMs received ART daily. After 13 to 14 weeks of ART, the PRI-724 treatment was initiated in the experimental group. Five RMs received 6 cycles of PRI-724 s.c. at 10 mg/kg/day, and 3 RMs received an uninterrupted treatment of PRI-724 s.c. at 20 mg/kg/day for 12 weeks. The control group was maintained on ART only.

**FIG 2:**
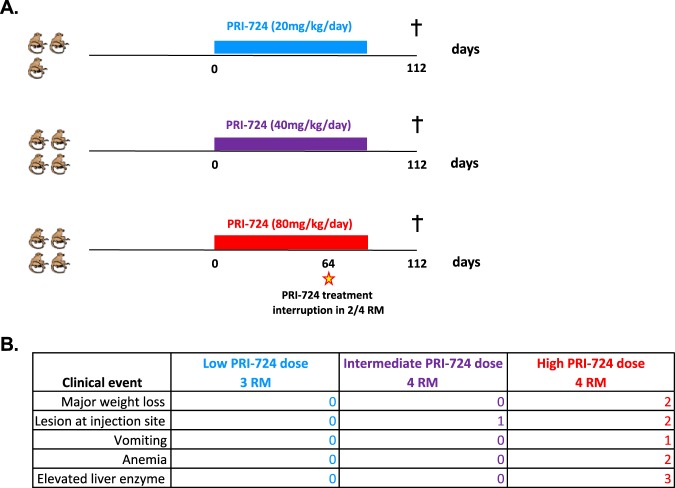
Toxicity study of PRI-724 in healthy rhesus macaques. (A) Study design. Eleven uninfected RMs received daily s.c. administration of PRI-724 for 12 weeks at a low dose (20 mg/kg/day for 3 RMs), intermediate dose (40 mg/kg/day for 4 RMs), and high dose (80 mg/kg/day for 4 RMs). The animals were monitored clinically, and frequent blood draws were collected to assess complete blood count and serum chemistries. (B) Adverse events per dose group.

**FIG 3:**
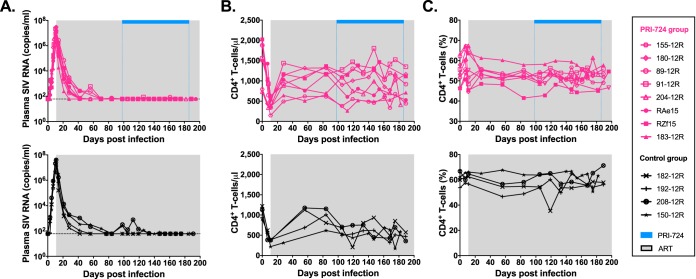
Virological and immunological parameters in PRI-724-treated and control ART-suppressed SIV-infected RMs. (A) Longitudinal assessment of plasma SIV RNA levels. Dotted lines represent the limit of detection of the assay. (B) Longitudinal assessment of the peripheral CD4^+^ T cell count. (C) Longitudinal assessment of the frequency of peripheral CD4^+^ T cells. PRI-724-treated RMs are depicted in pink, with open symbols representing RMs treated with PRI-724 at 10 mg/kg/day and filled symbols representing RMs treated with PRI-724 at 20 mg/kg/day. The control RMs are depicted in black. Gray shading represents the period of ART administration. Blue bars represent the period of PRI-724 injections.

Twelve Indian rhesus macaques (RMs), including 5 males and 7 females, were infected intravenously (i.v.) with 10^3^ 50% tissue culture infective dose (TCID_50_) of SIV_mac251_. Starting at day 11 postinfection (p.i.), all 12 animals were initiated on triple ART consisting of two reverse transcriptase inhibitors (tenofovir [PMPA] and emtricitabine [FTC]) and one integrase inhibitor (dolutegravir [DTG]). After 13 to 14 weeks on ART and a plasma viral load suppression of <80 copies/ml for at least 4 weeks, 8 RMs additionally received the CBP/β-catenin inhibitor PRI-724, while the 4 remaining RMs were maintained on ART only and served as controls (Fig. 1). Among the PRI-724-treated group, 5 RMs received 6 cycles (1 week on/1 week off) of PRI-724 at 10 mg/kg/day administered subcutaneously (s.c.). Based on results from a concurrent dose-ranging study (Fig. 2), an additional 3 RMs received 12 weeks of uninterrupted PRI-724 at 20 mg/kg/day s.c., a dose that was found to be safe in healthy RMs. As shown in Fig. 3A, following experimental infection with SIV_mac251_, the twelve RMs experienced a rapid, exponential increase in viremia, reaching levels of 10^6^ to 10^8^ SIV RNA copies/ml plasma. ART initiated at day 11 postinfection drastically reduced plasma viral loads to below the assay limit of detection after 3 to 10 weeks of treatment.

### Safety profile of PRI-724 administration in ART-suppressed SIV-infected RMs.

**FIG 4:**
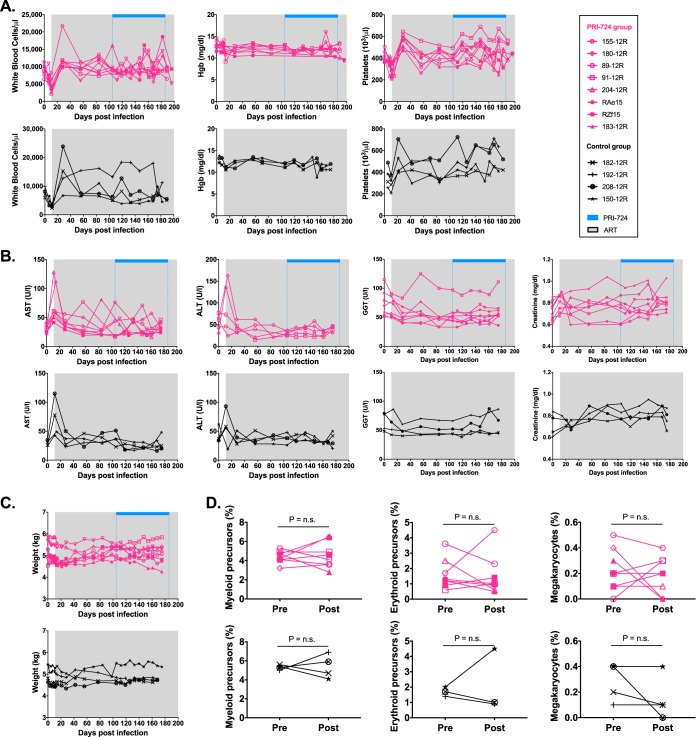
PRI-724 safety data in ART-suppressed SIV-infected RMs. Longitudinal assessment of (A) complete blood counts, (B) serum chemistries, and (C) weight in ART-treated SIV-infected RMs treated with PRI-724 compared to those of the controls. PRI-724-treated RMs are depicted in pink (open symbols, PRI-724 at 10 mg/kg/day; filled symbols, PRI-724 at 20 mg/kg/day) and control RMs in black. Gray shading represents the period of ART administration. Blue bars represent the period of PRI-724 injections. (D) Percentage of myeloid precursors, erythroid precursors, and megakaryocytes in the BM pre- and post-administration of PRI-724 (pink symbols) and at equivalent time points for the control RMs (black symbols). Preadministration samples were collected at weeks 15 to 16 p.i., immediately prior to the first dose of PRI-724, and postadministration samples were collected at weeks 27 to 28 p.i.

We first examined clinical and laboratory parameters to assess the safety of PRI-724 administration in this preclinical setting. PRI-724 administration did not affect plasma viral loads, which remained undetectable for the duration of treatment (Fig. 3A). Depletion of circulating CD4^+^ T cells was observed in all RMs during acute SIV infection, followed by a partial reconstitution of peripheral CD4^+^ T cell absolute count and frequency on ART and stable levels during PRI-724 treatment (Fig. 3B and C). Longitudinal assessment of hematological parameters, such as white blood cell (WBC) count, hemoglobin level, and platelet count, showed no significant difference between PRI-724-treated and control groups (Fig. 4A). Liver and kidney functions did not appear to be adversely impacted by PRI-724 treatment, based on stable serum levels of aspartate transaminase (AST), alanine transaminase (ALT), gamma-glutamyl transferase (GGT), and creatinine (Fig. 4B), again with no significant differences observed between treatment and control groups. Importantly, PRI-724-treated animals did not experience any clinical adverse events and, despite frequent sedation and blood collections, animal weights did not significantly fluctuate over the course of the treatment period (Fig. 4C). Similar results were found in healthy RMs given PRI-724 at both 20 mg/kg/day and 40 mg/kg/day s.c.; however, animals who received PRI-724 at a dose of 80 mg/kg/day s.c. developed significant weight loss, elevations in liver enzymes, and anemia. Intermediate and high dosing regimens were also associated with small subcutaneous abscesses at the injection sites (Fig. 2B).

Pharmacological modulation of the Wnt/β-catenin pathway could potentially affect trilineage hematopoiesis in the bone marrow. To assess for this potential consequence of PRI-724 treatment, we collected bone marrow at selected time points for pathological review. Overall bone marrow cellularity was unaffected by PRI-724, and trilineage hematopoiesis appeared normal, with no significant differences in the frequency of megakaryocytes, myeloid, or erythroid precursors pre- and post-PRI-724 treatment (Fig. 4D). Taken together, these results suggest that PRI-724 can be safely administered at doses of 10 to 20 mg/kg/day in ART-treated SIV-infected RMs.

### PRI-724 restricts SCM and CM T cell proliferation.

**FIG 5:**
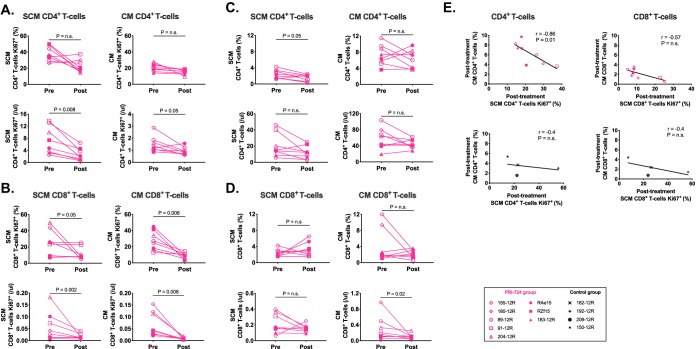
Flow cytometric assessment of long-lived memory T cells from PRI-724-treated ART-suppressed SIV-infected RMs. Frequencies (top panels) and absolute count (bottom panels) of (A) Ki67 expression in CD4^+^ T cells, (B) Ki67 expression in CD8^+^ T cells, (C) subsets of CD4^+^ T cells, and (D) subsets of CD8^+^ T cells pre- and postadministration of PRI-724. Preadministration samples were collected at weeks 15 to 16 p.i., immediately prior to the first dose of PRI-724, and postadministration samples were collected at weeks 27 to 28 p.i. (E) Correlations between Ki67 expression and cell subset frequencies postadministration of PRI-724 (pink symbols) and at equivalent time points for control RMs (black symbols). Open symbols represent RMs treated with PRI-724 at 10 mg/kg/day, and filled symbols represent RMs treated with PRI-724 at 20 mg/kg/day.

The predicted impact of inhibition of the CBP/β-catenin interaction is a reduction in proliferation/self-renewal of cells utilizing this pathway. To assess the effect of PRI-724 administration on T cell proliferation *in vivo*, we examined the frequency and absolute count of different subsets of CD4^+^ and CD8^+^ memory T cells in peripheral blood before and after treatment with PRI-724, as well as their level of expression of the proliferation marker Ki67. Remarkably, a decrease in the absolute count of SCM and CM CD4^+^ T cells expressing Ki67 was observed after PRI-724 treatment (Fig. 5A, bottom panels; *P* = 0.008 and *P* = 0.05). Similarly, the SCM and CM CD8^+^ T cells expressing Ki67 declined following PRI-724 treatment, as shown by the frequency (Fig. 5B, top panels; *P* = 0.05 and *P* = 0.008) and absolute count (Fig. 5B, bottom panels; *P* = 0.002 and *P* = 0.008). No significant changes were seen in the control group over the same period of time. Limited modulation of the levels of long-lived memory T cells was observed following PRI-724 treatment with a decrease in the frequency of SCM CD4^+^ T cells and absolute count of CM CD8^+^ T cells (Fig. 5C and D; *P* = 0.05, and *P* = 0.02). After PRI-724 treatment, a negative correlation was observed between proliferation of SCM CD4^+^ T cells and frequency of CM CD4^+^ T cells (*r* = −0.86, *P* = 0.01, Fig. 5E), showing that the PRI-724-related reduction in SCM CD4^+^ T cell proliferation was associated with an increase in the frequency of progeny CM CD4^+^ T cells. These results suggest that inhibiting the CBP/β-catenin interaction both reduces the self-renewal of long-lived memory CD4^+^ T cells and promotes their differentiation into shorter-lived cells.

### PRI-724 promotes a differentiation-enriched transcriptome in CM and SCM CD4^+^ T cells.

**FIG 6:**
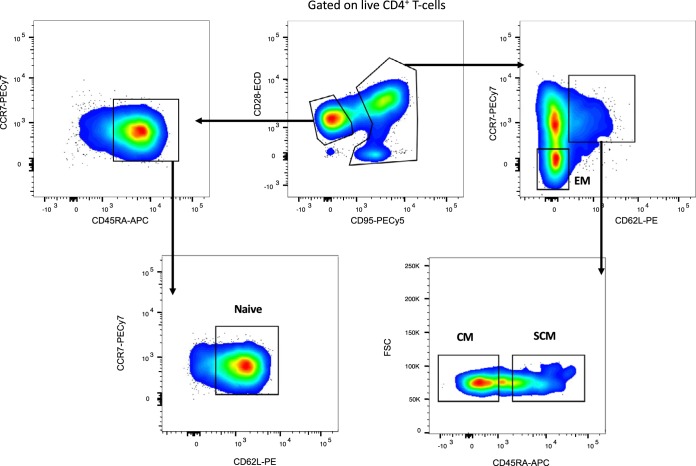
Gating strategy for the cell sorting of CD4^+^ T cell subsets. Representative fluorescence-activated cell sorting (FACS) dot plots showing the gating strategy used for the cell sorting of peripheral blood naive, SCM, CM, and EM CD4^+^ T cells.

**FIG 7:**
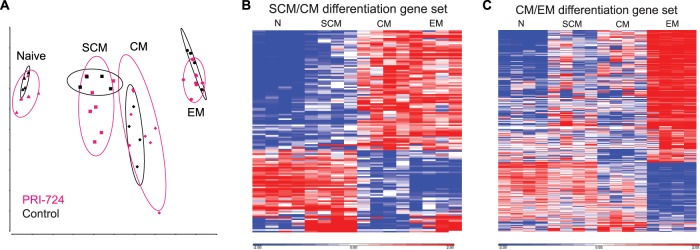
Whole-transcriptome analysis of circulating CD4^+^ T cell subpopulations in PRI-724-treated and control ART-suppressed SIV-infected RMs. (A) Principal component analysis of the transcriptome of subsets of naive, SCM, CM, and EM CD4^+^ T cells sorted by FACS from the peripheral blood postadministration of PRI-724 (pink symbols) and at equivalent time points for control RMs (black symbols). (B) Heat map of the genes differentially expressed in CM compared to SCM CD4^+^ T cells of the control RMs (at least 2-fold differential expression; adjusted *P* value of 0.05). Genes are shown in Data Sets S1, S2, and S3. (C) Heat map of the genes differentially expressed in CM compared to EM CD4^+^ T cells of the control RMs (at least 2-fold differential expression; adjusted *P* value of 0.05). Genes are shown in Data Sets S4, S5, and S6.

To define the impact of CBP/β-catenin interaction inhibition on CD4^+^ T cells, we assessed the transcriptional profile of populations of naive, SCM, CM, and EM CD4^+^ T cells sorted from the peripheral blood of the 8 experimental RMs after PRI-724 treatment and at an equivalent time point for the 4 control RMs (gating strategy shown in Fig. 6). We used RNA sequencing (RNA-Seq) to determine if PRI-724 treatment modified the transcriptomes of sorted CM and SCM CD4^+^ T cells to a differentiation-enriched state. Principal component analysis (PCA) demonstrated that the global transcriptomic profile of each cell subset was clearly distinguishable, thus validating our sorting strategy and the sensitivity and accuracy of our approach (Fig. 7A). Of note, the relative distances between sorted cell subsets showed that SCM and CM CD4^+^ T cells were more closely related to each other in their gene expression profile than to the other cell populations, confirming previous results obtained with CD8^+^ T cells (29). PRI-724-treated and control groups were transcriptionally similar within the naive and EM CD4^+^ T cell subsets, but they showed more variation within the SCM and CM CD4^+^ T cell populations, suggesting that the effect of PRI-724 was specific to these cell populations.

We next sought to examine the transcriptomic data for evidence that PRI-724 was able to confer to SCM or CM cells an expression profile consistent with differentiation. We first defined a set of genes that could discriminate SCM from CM cells by contrasting expression profiles in our untreated animals and identifying transcripts that were statistically overexpressed in SCM relative to CM T cells (defined as a false discovery rate [FDR] of <0.05 and a fold change of >2). The genes that discriminate between SCM and CM T cells are depicted in Fig. 7B, with naive and EM expression included for reference. We termed this set the “SCM/CM differentiation gene set.” We similarly defined a “CM/EM differentiation gene set” by contrasting CM with EM cells from untreated animals using the same gene-filtering criteria (Fig. 7C).

**FIG 8:**
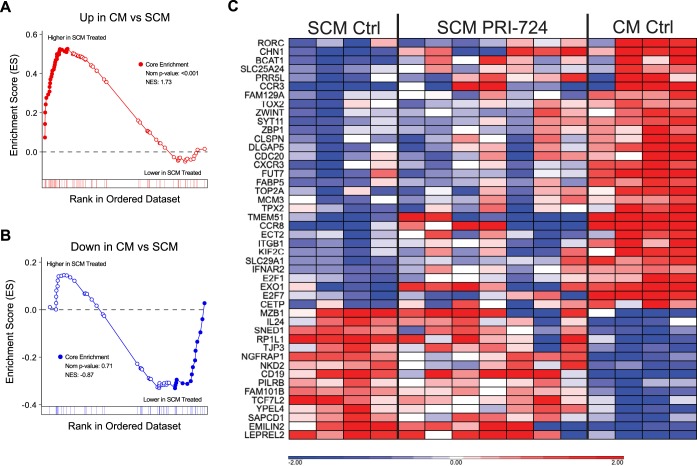
Expression of the SCM/CM differentiation gene set in SCM CD4^+^ T cells from PRI-724-treated ART-suppressed RMs. (A and B) Gene set enrichment plots of the “Up” and “Down” genes from the SCM/CM differentiation gene set plotted by rank on a contrast of gene expression profiles from PRI-724-treated cells (left side of GSEA plots) versus profiles from untreated cells (right side of GSEA plots). The enrichment score (*y* axis) is plotted by each gene’s individual rank (*x* axis); bars below the *x* axis indicate individual gene ranks in the whole data set. Genes in the leading edge (contributing the most to the enrichment score) are shown as filled symbols. (C) Heat map of leading-edge genes from the “up” and “down” gene sets showing their relative expression in PRI-724-treated SCM CD4^+^ T cells and untreated SCM and CM CD4^+^ T cells sorted from the peripheral blood of ART-suppressed SIV-infected RMs. Genes are shown in Data Sets S7, S8, and S9.

To test the hypothesis that PRI-724 treatment resulted in systematic upregulation of differentiation genes in SCM T cells from treated animals, we compared the transcriptomes of SCM T cells from the PRI-724-treated animals directly to those of SCM and CM T cells from untreated animals using gene set enrichment analysis (GSEA). Genes that we had identified as being upregulated in CM T cells compared to SCM T cells (“Up in CM vs SCM”) were significantly enriched in the SCM T cells of PRI-724-treated animals (FDR < 0.001; Fig. 8A). However, genes that we had identified as being downregulated in CM compared to SCM (“Down in CM vs SCM”) were not significantly enriched in the SCM of the PRI-724-treated animals (Fig. 8B). Manual inspection of the leading-edge genes in the “up” and “down” SCM/CM differentiation gene sets showed a similar effect (Fig. 8C). While variation exists between animals, this heat map provides a visual representation of the intermediate profile of cells transitioning from one differentiation stage to another following PRI-724 treatment.

**FIG 9:**
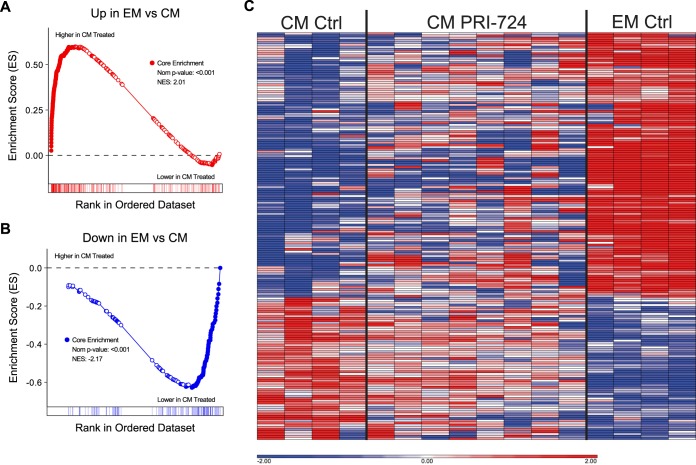
Expression of the CM/EM differentiation gene set in CM CD4^+^ T cells from PRI-724-treated ART-suppressed RMs. (A and B) Gene set enrichment plots of the “Up” and “Down” genes from the CM/EM differentiation gene set plotted by rank on a contrast of gene expression profiles from PRI-724-treated cells (left side of GSEA plots) versus profiles from untreated cells (right side of GSEA plots). The enrichment score (*y* axis) is plotted by each gene’s individual rank (*x* axis); bars below the *x* axis indicate individual gene ranks in the whole data set. Genes in the leading edge (contributing the most to the enrichment score) are shown as filled symbols. (C) Heat map of leading-edge genes from the “up” and “down” gene sets showing their relative expression in PRI-724-treated CM CD4^+^ T cells and untreated CM and EM CD4^+^ T cells sorted from the peripheral blood of ART-suppressed SIV-infected RMs. Genes are shown in Data Sets S10, S11, and S12.

A similar approach was used to assess CM CD4^+^ T cells from PRI-724-treated versus control RMs, with both “up” CM/EM differentiation genes and “down” CM/EM differentiation genes being statistically significantly enriched or reduced, respectively, in CM T cells from PRI-724-treated animals (Fig. 9A and B, *P* < 0.001 for both comparisons). The leading-edge gene heat map (Fig. 9C) again demonstrates a transcriptomic profile of CM CD4^+^ T cells from PRI-724-treated RMs that is intermediate between those of CM and EM CD4^+^ T cells of controls, suggesting that these cells were captured during a period of differentiation.

### PRI-724 did not reduce the level of cell-associated SIV DNA in memory CD4^+^ T cells.

**FIG 10:**
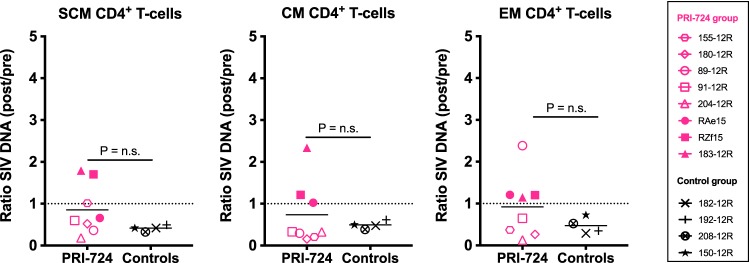
Assessment of SIV DNA levels in memory CD4^+^ T cells of PRI-724-treated ART-suppressed SIV-infected RMs. The ratios of cell-associated SIV DNA levels post-administration of PRI-724 relative to preadministration and at equivalent time points for control RMs were calculated in subsets of SCM CD4^+^ T cells, CM CD4^+^ T cells, and EM CD4^+^ T cells. PRI-724-treated RMs are depicted in pink (open symbols, PRI-724 at 10 mg/kg/day; filled symbols, PRI-724 at 20 mg/kg/day) and control RMs in black. Preadministration samples were collected at weeks 15 to 16 p.i., immediately prior to the first dose of PRI-724, and postadministration samples were collected at weeks 27 to 28 p.i.

We then evaluated the impact of the pharmacological inhibition of CBP/β-catenin interaction on the persistent viral reservoir by measuring by PCR the frequency of infection of CD4^+^ T cell subsets sorted by fluorescence-activated cell sorting (FACS) from the peripheral blood. Most animals from both PRI-724-treated and control groups experienced a decrease in cell-associated SIV DNA in the different subsets of CD4^+^ T cells at weeks 27 to 28 compared to weeks 15 to 16 after infection, likely related to the expected reduction in virus persistence due to ART. We thus compared the ratios of cell-associated SIV DNA before (weeks 15 to 16) and after PRI-724 treatment (weeks 27 to 28) or over a similar time period in the controls (Fig. 10). The ratios were similar for each subset (0.15- to 2.3-fold change), suggesting that PRI-724 treatment alone did not significantly reduce the viral reservoir in ART-suppressed SIV-infected RMs.

## DISCUSSION

Developing novel strategies to eliminate viral reservoirs that could lead toward a cure or lifelong remission of HIV infection remains a key priority in HIV/AIDS research. The reservoir of latently infected memory CD4^+^ T cells that persists on long-term ART represents the main barrier to HIV cure. Recent studies establishing clonal expansion of HIV-infected cells as an important mechanism of reservoir maintenance suggest that reaching a functional cure will likely require interference with the proliferation of infected cells, particularly memory CD4^+^ T cell subsets with long life spans and important clonogenic properties (16, 18–21, 41).

Here, we tested an innovative approach aimed at reducing SIV persistence by inhibiting the self-renewal properties of the long-lived CM and SCM CD4^+^ T cells. We used the robust nonhuman primate model of SIV infection of RMs to evaluate a small molecule targeting an intracellular signaling pathway controlling cell proliferation. While Wnt inhibition initially raised toxicity concerns, PRI-724, has completed phase Ia clinical evaluation and has proven to be safe in humans (42–44). Previous work in healthy volunteers and oncology patients suggests that pharmacologic inhibition of CBP/β-catenin may be readily translatable to clinical studies in persons living with HIV. In the work described here, PRI-724 doses of 10 to 20 mg/kg/day were administered to 8 SIV-infected macaques and an additional 4 uninfected animals without adverse events. The concurrent dose-ranging study indicated that PRI-724 doses of up to 40 mg/kg/day may be safe in healthy macaques, but administration of 80 mg/kg/day of PRI-724 was associated with both clinical and laboratory abnormalities.

The administration of PRI-724 to ART-suppressed SIV-infected RMs induced a consistent decline in Ki67 expression in circulating SCM and CM CD4^+^ and CD8^+^ T cells. This result suggests that PRI-724 can reduce the proliferation of long-lived memory T cells *in vivo*. In line with this result, inhibition of CBP/β-catenin by PRI-724 has been associated with decreased expression of proliferation-related markers in hepatic stellate cells in a murine model of fibrosis (45). Similarly, other β-catenin inhibitors have been shown to reduce Ki67 expression in various cancer models (46–48). The antiproliferative capacities of PRI-724 are being further evaluated in several ongoing phase I/II trials in hematological malignancies, pancreatic cancer, and colon cancer. After PRI-724 treatment, a negative correlation was observed in the peripheral blood between proliferation of SCM CD4^+^ T cells and frequency of CM CD4^+^ T cells. In a highly dynamic system, the observations that the frequency and absolute count of CM cells remained stable while that of CM cells expressing Ki67 declined indicates that pharmacologic inhibition of SCM self-renewal with PRI-724 is also associated with the promotion of SCM differentiation into the less-differentiated CM population.

Using the more comprehensive approach of RNA-Seq, we showed that treatment with PRI-724 led to a consistent programming of gene expression profiles of less-differentiated memory CD4^+^ T cells toward that of more-differentiated cells, with SCM CD4^+^ T cells exhibiting a transcriptomic profile intermediate to those of SCM and CM CD4^+^ T cells and CM CD4^+^ T cells exhibiting a transcriptomic profile intermediate to those of CM and EM CD4^+^ T cells. Therefore, by using the complementary approaches of flow cytometry and transcriptomic analyses, we demonstrate that interference with the CBP/β-catenin interaction *in vivo* results in restricted proliferation and enhanced differentiation of long-lived memory CD4^+^ T cells. As recent studies using mathematical modeling suggest that antiproliferative therapies used in combination with ART could reduce the time needed to reach a functional cure (41, 49), the identification of drugs that can safely reduce the proliferation of latently infected cells may be an important step forward.

Pharmacological targeting of CBP/β-catenin did not induce virus reactivation, as shown by the maintenance of plasma viral loads below the limit of detection following PRI-724 administration in ART-treated SIV-infected RMs. This result was expected, as our approach represents a paradigm shift away from strategies that require latency reversal, i.e., the “shock and kill” approach (50–52). Instead, we chose to target the most long-lasting, stem cell-like component of the viral reservoir that can indefinitely repopulate latently infected CD4^+^ T cells in HIV-infected individuals. Our results indicate that long-lived memory CD4^+^ T cell differentiation can occur without virus reactivation. While PRI-724 is not specific to SCM and CM T cells (as β-catenin is active in other cells, including nonimmunological stem cells), it should be noted that this intervention is more specific, in terms of cellular targets, than the first generation of latency-reversing agents, such as histone deacetylase (HDAC) inhibitors, which have been extensively studied in humans as potential approaches to reduce the virus reservoir. Furthermore, trilineage hematopoiesis was preserved in PRI-724-treated animals, alleviating concern regarding its potential impact on normal cellular development at the tested doses.

ART duration was limited to 13 to 14 weeks before PRI-724 treatment in this pilot experiment, and the decline of cell-associated SIV DNA levels in circulating CD4^+^ T cell subsets in both PRI-treated and control groups was thus expected as a function of time on ART irrespective of the experimental intervention. Unbiased analyses of the ratios of cell-associated DNA levels preadministration relative to postadministration of PRI-724 also did not reveal a difference between groups, thus suggesting a lack of effect of 12 weeks of PRI-724 treatment alone on the viral reservoir in our model. Due to our focus on estimating the size of the persistent SIV reservoir in subsets of memory CD4^+^ T cells, we were restricted to the assessment of total cell-associated SIV DNA with available cell numbers. We recognize that this assay measures both intact and defective proviruses, thus overestimating the size of the replication-competent SIV reservoir. Future work using this antiproliferation, prodifferentiation approach should be designed to utilize quantitative virus outgrowth or intact proviral DNA assays (53) to understand the impact of the intervention on the replication-competent reservoir. Targeting additional pathways regulating the proliferation or differentiation of memory CD4^+^ T cells, such as Notch or Hedgehog signaling pathways, is the subject of these ongoing studies. Using these approaches in combination with latency reversal agents and immunotherapeutics may be warranted to effectively reduce the size of the viral reservoir. A theoretically promising strategy would be to selectively promote the differentiation of latently infected memory CD4^+^ T cells into shorter-lived cells that are more prone to die and are more susceptible to virus reactivation and immune-mediated killing (54).

In conclusion, our study established a safe dosage of the CBP/β-catenin antagonist PRI-724 in ART-suppressed SIV-infected RMs. We showed that targeting Wnt signaling pathway can restrict the proliferation and promote the differentiation of long-lived memory CD4^+^ T cells. To our knowledge, this is the first study targeting the self-renewal properties of long-lived latently infected CD4^+^ T cells as a key component of interventions aimed at reducing HIV/SIV persistence. This approach actively considers the structural and developmental heterogeneity of latently infected memory CD4^+^ T cells, focusing a specific molecular strategy on a core feature of the viral reservoir that represents a substantial barrier to virus eradication. A transient block to the proliferation of long-lived latently infected memory CD4^+^ T cells via selective inhibition of stem cell-like signaling pathways may represent a necessary and complementary approach to strategies directly targeting latent HIV.

## MATERIALS AND METHODS

### Animals and infection.

Twelve Indian RMs (Macaca mulatta) with exclusion of Mamu B*08- and B*17-positive animals, were enrolled in this study. They were all infected i.v. with 10^3^ TCID_50_ of SIV_mac251_ and treated with ART before receiving or not receiving the experimental PRI-724 treatment. Concurrently, a PRI-724 dose-ranging study was performed in 11 healthy, uninfected Indian RMs. All animals were housed at the Yerkes National Primate Research Center (Atlanta, GA) and treated in accordance with Emory University and Yerkes National Primate Research Center Institutional Animal Care and Use Committee regulations.

### Antiretroviral therapy and PRI-724 treatment.

The 12 SIV-infected RMs were treated with a potent 3-drug ART regimen initiated 11 days postinfection. The preformulated ART cocktail contained two reverse transcriptase inhibitors, 20 mg/ml tenofovir (TFV), and 40 mg/ml emtricitabine (FTC), plus 2.5 mg/ml of the integrase inhibitor dolutegravir (DTG). This ART cocktail was administered once daily at 1 ml/kg body weight via the subcutaneous (s.c.) route. PRI-724 was administered s.c. for up to 12 weeks at concentrations ranging from 10 to 80 mg/kg/day.

### Sample collection and processing.

EDTA-anticoagulated blood samples were collected regularly and used for a complete blood count, routine chemical analysis, and immunostaining, with plasma separated by centrifugation within 1 h of phlebotomy. Peripheral blood mononuclear cells (PBMCs) were prepared by density gradient centrifugation and cryopreserved at −80°C until use.

### Immunophenotyping by flow cytometry.

Multicolor flow cytometric analysis was performed on whole blood or a cell suspension using predetermined optimal concentrations of the following fluorescently conjugated monoclonal antibodies (MAbs): CD3-APC-Cy7 (clone SP34-2), CD95-PE-Cy5 (clone DX2), *K_i_*-67-AF700 (clone B56), HLA-DR-PerCP-Cy5.5 (clone G46-6), CCR7-FITC (clone 150503), CCR5-APC (clone 3A9), and CD45RA-PECy7 (clone L48) from BD Biosciences; CD8-BV711 (clone RPA-T8), CD4-BV650 (clone OKT4), and PD-1-BV421 (clone EH12.2H7) from BioLegend, and CD28-ECD (clone CD28-2) from Beckman Coulter. Flow cytometric acquisition and analysis of samples were performed on at least 100,000 events on an LSR II flow cytometer driven by the FACSDiva software package (BD Biosciences). Analyses of the acquired data were performed using FlowJo version 10.0.4 software (TreeStar).

### Cell sorting.

After isolation, cells were resuspended in phosphate-buffered saline (PBS) containing 2 mM EDTA and spun for the removal of contaminating platelets. Prior to sorting, peripheral CD4^+^ T cells were enriched with the use of magnetic beads and column purification (Miltenyi Biotec). Enriched peripheral CD4^+^ T cells were then stained with previously determined volumes of the following fluorescently conjugated MAbs: CD3-APC-Cy7 or CD3-AF700 (clone SP34-2), CCR7-PE-Cy7 (clone 3D12), CD8-APC-Cy7 (clone SK1), CD45RA-APC (clone 5H9), CD95-PE-Cy5 (clone DX2), and CD62L-PE (clone SK11) from BD Bioscience; CD28-ECD (clone CD28.2) from Beckman Coulter, and CD4-BrilliantViolet650 (clone OKT4) and CD8-BV421 (clone RPA-T8) from BioLegend. Circulating populations for sorting were defined as follows: naive, CD45RA^+^ CCR7^+^ CD95^−^ SCM, CD45RA^+^ CCR7^+^ CD95^+^ CD28^+^ CD62L^+^; CM, CD45RA^−^ CD95^+^ CCR7^+^ CD62L^+^; and EM, CD95^+^ CCR7^−^. Sorting was performed on a FACSAria LSR II (BD Biosciences) equipped with FACSDiva software.

### Plasma RNA and cell-associated DNA viral quantification.

Plasma viral quantification was performed in the core virology laboratory of the Emory Center for AIDS Research as described previously (55). Frozen cell pellets were lysed with proteinase K (100 μg/ml in 10 mM Tris-HCl [pH 8]) for 1 h at 56°C. Quantification of SIV_mac_ *gag* DNA was performed by quantitative PCR using the 5′ nuclease (TaqMan) assay with an ABI7500 system (PerkinElmer Life Sciences). The sequence of the forward primer for SIV_mac_ *gag* was 5′-GCAGAGGAGGAAATTACCCAGTAC-3′, the reverse primer sequence was 5′-CAATTTTACCCAGGCATTTAATGTT-3′, and the probe sequence was 5′-6-carboxyfluorescein-(FAM) TGTCCACCTGCCATTAAGCCCGA-6-carboxytetramethylrhodamine (TAMRA)-3′. Cell lysate (7.5 μl) was mixed in a 50-μl reaction mixture containing 1× Platinum buffer, 3.5 mM MgCl_2_, 0.2 mM deoxynucleoside triphosphate (dNTP), 200 nM primers, 150 nM, probe, and 2 U Platinum *Taq*. For cell number quantification, quantitative PCR was performed simultaneously for monkey albumin gene copy number. The sequence of the forward primer for albumin was 5′-TGCATGAGAAAACGCCAGTAA-3′, the reverse primer sequence was 5′-ATGGTCGCCTGTTCACCAA-3′, and the probe sequence was 5′-AGAAAGTCACCAAATGCTGCACGGAATC-3′ (56). The reactions were performed on a 7500 real-time PCR system (Applied Biosystems) with the following thermal program: 5 min at 95°C, followed by 40 cycles of denaturation at 95°C for 15 s and annealing at 60°C for 1 min. The limit of detection for this assay is 60 copies per ml of plasma.

### RNA-Seq analyses.

RNA-Seq analysis was conducted at the Yerkes Nonhuman Primate Genomics Core Laboratory (http://www.yerkes.emory.edu/nhp_genomics_core/). RNA was purified using Qiagen Micro RNeasy columns, and RNA quality was assessed using an Agilent Bioanalyzer instrument. Total RNA (10 ng) was used as input for mRNA amplification using 5′ template-switch PCR with the Clontech SMART-Seq v4 Ultra Low Input RNA kit according to the manufacturer’s instructions. Amplified mRNA was fragmented and appended with dual-indexed barcodes using Illumina Nextera XT DNA library prep kits. Libraries were validated by capillary electrophoresis on an Agilent 4200 TapeStation, pooled, and sequenced on an Illumina HiSeq 3000 sequencer using 151 bases (single end) at an average read depth of 23 million reads.

RNA-Seq reads were aligned to the MacaM version 7.8 assembly of the Indian RM genome (available at https://www.unmc.edu/rhesusgenechip/index.htm). Alignment was performed with STAR version 2.5.2b (https://github.com/alexdobin/STAR), and transcript abundance was estimated using htseq-count v0.6.1p1 (http://htseq.readthedocs.io/). Transcript abundance was estimated during the alignment using the method of htseq-count (http://htseq.readthedocs.io/) Read counts were normalized and differential expression analysis was performed with DESeq2 (https://bioconductor.org/packages/release/bioc/html/DESeq2.html). GSEA was performed using the desktop module available from the Broad Institute (https://www.broadinstitute.org/gsea/).

### Statistical analyses.

Comparisons between pre-administration and post-administration of PRI-724 or equivalent time points for control time points were determined by a paired Wilcoxon matched-pair signed-rank test. Ratios of pretreatment to posttreatment data were compared using an unpaired Mann-Whitney U test. Correlations were determined using the non-Gaussian Spearman correlation. Significance was attributed at *P* values of ≤0.05. Analyses were done using GraphPad Prism version 6.0. RNA-Seq differential expression analysis and statistical analyses were performed using the DESeq2 package.

## Supplementary Material

Supplemental file 1
